# Central lymph node ratio is an important recurrence prognostic factor for pediatric differentiated thyroid cancer

**DOI:** 10.3389/fendo.2024.1290617

**Published:** 2024-07-02

**Authors:** Caixin Qiu, Shipeng Wu, Jiehua Li

**Affiliations:** ^1^ Department of Gastroenterology and Gland Surgery, The First Affiliated Hospital of Guangxi Medical University, Nanning, Guangxi Zhuang Autonomous Region, China; ^2^ Department of First Clinical Medical College, Guangxi Medical University, Nanning, Guangxi Zhuang Autonomous Region, China; ^3^ Department of Thyroid and Breast Surgery, Yulin First People’s Hospital, Yulin, Guangxi Zhuang Autonomous Region, China

**Keywords:** lymph node ratio, pediatric, thyroid cancer, recurrence, risk stratification

## Abstract

**Background:**

The current risk stratification methods for Pediatric Differentiated Thyroid Carcinoma (DTC) are deemed inadequate due to the high recurrence rates observed in this demographic. This study investigates alternative clinicopathological factors, specifically the Central Lymph Node Ratio (CLNR), for improved risk stratification in pediatric DTC.

**Methods:**

A retrospective review of 100 pediatric DTC patients, aged 19 or younger, treated between December 2012 and January 2021 at the First Affiliated Hospital of Guangxi Medical University was conducted. Clinicopathological variables were extracted, and univariate logistic regression identified factors correlated with recurrence. Kaplan-Meier (KM) survival analysis and subsequent statistical tests were used to assess the significance of these factors.

**Results:**

The CLNR, with a cutoff value of 77.78%, emerged as a significant predictor of recurrence. Patients with a CLNR above this threshold had a 5.467 times higher risk of recurrence. The high CLNR group showed a higher proportion of male patients, clinically lymph node positivity (cN1), and extrathyroidal extension (ETE) compared to the low-risk group (p<0.05).

**Conclusion:**

CLNR is a valuable predictor for recurrence in pediatric DTC and aids in stratifying patients based on Recurrence-Free Survival (RFS). For patients with a high CLNR, aggressive iodine-131 therapy, stringent TSH suppression, and proactive postoperative surveillance are recommended to mitigate recurrence risk and facilitate timely detection of recurrent lesions.

## Introduction

Thyroid cancer ranks as the fourth most prevalent cancer in adults, yet it accounts for a mere 3% of all pediatric cancers (1, 2). Despite this, the incidence of pediatric thyroid cancer is increasing (3). Within this demographic, differentiated thyroid cancers (DTC) constitute 95% of cases, with papillary thyroid cancer predominating at 90% of DTC cases and follicular cancer comprising the remainder (4–6). Nonetheless, pediatric DTC exhibits a lower mortality rate than its adult counterpart (7, 8). The AJCC TNM system, 8th edition, solely considers the anatomic location of metastatic lymph nodes for nodal staging, delineating pN1a (central neck metastasis) and pN1b (lateral neck) (9). The 2015 ATA guidelines for pediatric DTC indicate that the TNM classification limits pediatric patients to stage I (no distant metastasis) or stage II (with distant metastasis) (6). Recognizing the TNM system’s limitations in stratifying pediatric DTC with high recurrence rates, the ATA has developed a risk stratification system categorizing children into low, intermediate, and high-risk groups. However, the criteria for defining intermediate risk, particularly regarding the number of lymph nodes for extensive N1a, remain ambiguous. Consequently, further investigation is warranted to establish the optimal threshold for metastatic lymph nodes. Additionally, the utility of this risk system in a large cohort has yet to be validated (10). Recent research has underscored the lymph node ratio (LNR) as a more reliable prognostic indicator for recurrence in adult thyroid cancer than TNM staging (9). Jeon et al. have also shown that the number of metastatic lymph nodes and extrathyroidal extension are predictive of recurrence in pediatric DTC (10). However, the role of LNR as a recurrence predictor in pediatric DTC has not been thoroughly explored. Our study aims to identify statistically significant variables influencing recurrence and recurrence-free survival in pediatric DTC by analyzing clinicopathological features, including lymph node ratio, number, and location of metastatic lymph nodes.

## Materials and methods

Guidelines from the American Thyroid Association and current research on pediatric differentiated thyroid carcinoma (DTC) suggest that the age threshold of 18 years for defining pediatric DTC is not evidence-based (6). The majority of pediatric DTC research and guidelines encompass patients under the age of 21 (11–13). Given the ongoing debate regarding the age criteria for pediatric DTC, our study defines the pediatric population as individuals under 19 years of age. Our investigation includes a comprehensive review of all pediatric DTC cases managed at the First Affiliated Hospital of Guangxi Medical University from December 2012 to January 2021, focusing on patients aged 19 or younger at the time of treatment.

The patients’ electronic medical records were reviewed to compile data on demographics, clinical cervical lymph node status, distant metastasis, operative reports, surgical pathology, follow-up for recurrence or progression surveillance, and postoperative treatment, including radioactive iodine ablation (RAI).

Demographic data, including age and gender, were documented for each patient. Clinical lymph node status was ascertained through physical examination, ultrasound, computed tomography (CT), or magnetic resonance imaging (MRI) of the cervical lymph nodes. Patients with preoperative findings suggestive of lymph node metastasis were classified as clinically lymph node positive (cN1). Distant metastasis was assessed using preoperative chest CT or postoperative initial RAI scintigraphy, with synchronous metastasis defined as that identified before or within 6 months post-surgery, and metachronous metastasis as that found more than 6 months after surgery.

Pathology reports provided tumor location and size, along with data on metastatic and total lymph nodes. Additional recorded factors included lymphovascular invasion, tumor multifocality, Hashimoto thyroiditis (HT) presence, and gross (gETE) and micro-extrathyroidal extension (mETE) of the thyroid tumor. mETE was characterized by tumor invasion of the thyroid or perithyroidal tissue under microscopy (14), while gETE was defined by tumor extension into perithyroidal tissue on gross pathology (15). Extranodal extension in dissected lymph nodes was identified through macropathology or pathology reports indicating lymph node capsule invasion. Multifocality referred to the presence of two or more thyroid lesions, and bilateral disease indicated malignant lesions in both lobes. The lymph node ratio was calculated as the number of metastatic lymph nodes divided by the number of intraoperatively harvested lymph nodes, with the central lymph node ratio specific to the central compartment and the lateral lymph node ratio to the lateral neck compartment.

Data on the date of surgery, type of lymph node dissection (central or central plus lateral), extent of thyroidectomy, and intraoperative findings of the thyroid tumor and cervical lymph nodes were extracted from surgical records. The central lymph node compartment was delineated as the area bounded by the hyoid bone superiorly, the posterior sternal notch inferiorly, the middle part of the carotid sheath transversely, and the prevertebral fascia dorsally. Bilateral central lymph node dissection was indicated for malignant tumors in both lobes or the isthmus.

Lateral cervical lymph node enlargement on preoperative examination prompted fine needle aspiration biopsy (FNAB), with positive results leading to lateral neck lymph node dissection. If FNAB was not performed but intraoperative findings included a substantial number of metastatic central compartment lymph nodes along with enlarged lateral cervical lymph nodes, further lateral neck lymph node dissection was pursued. Modified lateral lymph node dissection, preserving the sternocleidomastoid muscle, internal jugular vein, and spinal accessory nerve, was performed based on central lymph node dissection, targeting levels II-V. Thyroidectomy ranged from total to subtotal and lobectomy. Total thyroidectomy was considered for malignant lesions in both thyroid lobes, extrathyroidal extension on gross intraoperative view, or intraoperative fast-frozen pathology revealing central lymph node metastasis greater than five. Lobectomy with isthmectomy was standard for unilateral malignant lesions; subtotal thyroidectomy was performed when one lobe had a malignant and the other a benign lesion. RAI was selected based on preoperative FNAB, intraoperative fast-frozen pathology, and gross pathology; patients meeting RAI criteria were ineligible for subtotal thyroidectomy or lobectomy with isthmectomy (RAI criteria included lymph node metastasis >5, distant metastasis, and extrathyroidal extension).

Postoperatively, patients underwent TSH suppression, with follow-up examinations every 3 months for the first postoperative year, including serum TSH and Thyroglobulin level testing, and neck ultrasound or CT. Thereafter, follow-ups occurred every six months for five years, then annually. The follow-up period was measured from the initial surgery to the last contact, ascertained through medical record review or direct patient contact. Recurrence-free survival (RFS) assessed our cohort, with recurrence categorized as local (confirmed by biopsy or surgery pathology in the neck) or distant (new lesions on CT or RAI scans outside the neck). Distinguishing true recurrence from undetected microscopic tumor growth on initial imaging is challenging. Our criteria for recurrence focused on identifying lesions not apparent on previous imaging but discovered in subsequent tests, defining these as recurrences.

The study was approved by the First Affiliated Hospital of Guangxi Medical University Institutional Review Board and conducted in accordance with the Declaration of Helsinki.

### Statistical analysis

Statistical analyses were conducted using R software version 4.2.1 and SPSS software version 25.0. Continuous variables are presented as median (range).

First, use the R packages ‘survminer’ and ‘survival’ to segment continuous variables by identifying cutoff values based on recurrence free survival (RFS). After determining the cutoff values for each variable, perform a univariate logistic regression analysis to examine the impact of all variables on recurrence as the outcome. Once statistically significant variables are identified, use Kaplan-Meier (KM) survival analysis to analyze the variable in question and plot the corresponding survival curves.

After identifying variables associated with RFS, we categorize patients based on these variables. Then, we perform inter-group comparisons using the Mann-Whitney U test and chi-squared test. Finally, for variables that show statistical significance in inter-group comparisons, we conduct a multivariate logistic regression analysis to predict the high-risk group. All tests were two-sided, with a p-value < 0.05 considered statistically significant.

## Results

Our study cohort comprised 100 patients with complete medical records, with a median age at diagnosis of 17 years. Females constituted 78% of the cohort. None had a prior cancer history, while 4% had received head/neck irradiation. Clinical lymph node positivity (cN1) was observed in 45% of patients. Classic papillary thyroid cancer (PTC) was the most common diagnosis (92%), followed by the follicular variant (5%), diffuse sclerosing variant (2%), and follicular thyroid carcinoma (FTC) (1%). Hashimoto thyroiditis (HT) coexisted in 10% of cases, and lymphovascular invasion was present in 11%. Multifocality was identified in 39% of patients, with bilateral disease and gross-extrathyroidal extension observed in 36%. Micro-extrathyroidal extension was found in 10% of patients. Tumor size varied, with 8% having tumors ≤1 cm, 36% having tumors >1 cm but ≤2 cm, 41% having tumors >2 cm but ≤4 cm, and 15% having tumors >4 cm. Central neck dissection was performed in 94 patients, with 87.23% exhibiting central lymph node metastasis (CLNM). The median number of harvested and metastatic central lymph nodes was 8 and 4, respectively. Lateral lymph node dissection was conducted in 56 patients, with 89.28% showing lateral lymph node metastasis (LLNM). The median number of harvested and metastatic lateral lymph nodes was 20 and 10, respectively. Extranodal extension was identified in 18% of cases. Total thyroidectomy (TT) was performed in 64% of patients, while lobectomy plus isthmectomy or subtotal thyroidectomy was conducted in 36%. Postoperative RAI was administered to 68% of the cohort. During a median follow-up period of 53.22 months (range 17.37–116.17 months), 13% of patients experienced recurrence, with seven patients having unilateral lateral cervical lymph node recurrence, three patients experiencing central lymph node recurrence, one patient exhibiting recurrence in both the central and lateral cervical lymph node regions, and two patients showing bilateral lateral cervical lymph node recurrence. Notably, there were no DTC-related fatalities, and no cases of disease progression were observed (**Table 1**).

**Table 1:** Baseline characteristics of pediatric patients with DTC.

Characteristic	Median (Range)	N (%)
Age at diagnosis(n=100)	17(7–19)	
Follow-up(months)	53.22(17.37–116.17)	
Sex		
Male		22(22%)
Female		78(78%)
history of previous cancer		
Absent Present		100(100%)
Present		0(0%)
history of head/neck irradiation		
Absent Present		96(96%)
Present		4(4%)
Diagnosis		
Classic PTC		92(92%)
fvPTC		5(5%)
dsvPTC		2(2%)
FTC		1(1%)
Lymphovascular invasion		
Absent		89(89%)
Present		11(11%)
cN1		
Absent		55(55%)
Present		45(45%)
Synchronous distant metastasis		
Absent		95
Present		5
Tumor length	2.2(0.3–8.1)	
≤1cm		8(8%)
1.1–2.0		36(36%)
2.1–4.0		41(41%)
>4		15(15%)
Bilateral disease		
Absent		64(64%)
Present		36(36%)
Extent of thyroid surgery		
TT		64(64%)
Less than Total thyroidectomy		36(36%)
Extent of lymph node dissection		
CLN dissection		38(38%)
CLN+LLN dissection		56(56%)
Not performed		6(6%)
Multifocality		
Absent		61(61%)
Present		39(39%)
Hashimoto thyroiditis		
Absent		80(80%)
Present		20(20%)
Gross-ETE		
Absent		64(64%)
Present		36(36%)
Micro-ETE		
Absent		90(90%)
Present		10(10%)
Extranodal extension		
Absent		82(82%)
Present		18(18%)
Postrperative RAI		
Absent		32(32%)
Present		68(68%)
Recurrence		
Absent		87(87%)
Present		13(13%)
CLNM*		
Absent		12(12.77%)
Present		82(87.23%)
LLNM		
Absent		6(10.71%)
Present		50(89.29%)
Number of harvested CLN(n=85) **	8(0–19)	
Number of metastatic CLN(n=85)	4(0–17)	
Number of harvested LLN(n=47)	20 (0–54)	
Number of metastatic LLN(n=47)	10(0–28)	
LLNR(n=47)	32.74% (0–100%)	
CLNR(n=85)	75% (0–100%)	

Classic PTC, classic papillary thyroid cancer; fvPTC, follicular variant papillary thyroid cancer; dsvPTC, diffuse sclerosing variant papillary thyroid cancer; FTC, follicular thyroid cancer; cN1, clinically lymph node positive; TT, total thyroidectomy; CLN, central lymph node; LLN, lateral lymph node; gross-ETE, gross-extrathyroidal extension; micro-ETE, micro-extrathyroidal extension; RAI, radioactive iodine ablation; CLNM, central lymph node metastasis; LLNM, lateral lymph node metastasis; CLNR, central lymph node ratio; LLNR, lateral lymph node ratio.

*Excluded 6 patients without central lymph node dissection.

**Excluded 6 patients without central lymph node dissection and 9 patients without detail information on the number of regional lymph node metastases.

**Figure 1:**
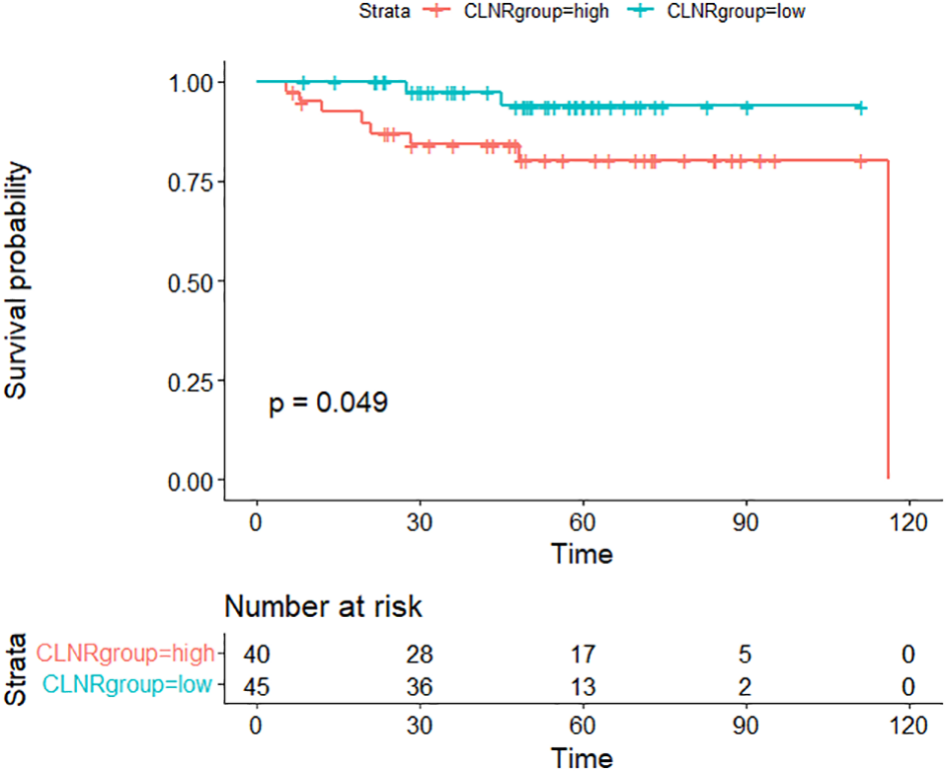
Comparison of recurrence-free survival (RFS) between CLNR high group (>77.78%) and CLNR low group (≦77.78%)(p<0.05).

Kaplan-Meier survival analysis revealed RFS rates of 95.9% at 1 year, 90.4% at 3 years, and 87.5% at 5 years. To ascertain variables significantly contributing to RFS, various factors were used to stratify RFS (**Table 2**). Univariate logistic regression analysis identified the central lymph node ratio (CLNR) as the sole statistically significant factor for recurrence (p=0.040). Subsequent Kaplan-Meier survival analysis confirmed the statistical significance of CLNR for RFS, as depicted in **Figure 1**. The determined CLNR cutoff value of 77.78% indicated that patients with a CLNR exceeding this threshold had a 5.467-fold higher likelihood of recurrence in pediatric DTC (HR=5.467, CI=1.082–27.609). Notably, the R packages ‘Survminer’ and ‘Survival’ facilitated the automatic identification of the optimal stratification threshold for RFS in continuous variables.

**Table 2:** Univariate logistic regression for recurrence.

Independent variable	Univariate	
	HR(95%CI)	p value
Gender (male/female)	0.872(0.216–3.522)	0.847
Age (≤13/>13)	1.071(0.844–1.359)	0.572
Subtype (classic PTC/others)	0.947(0.105–8.574)	0.962
Lymphovascular invasion (Y/N)	3.214(0.714–14.476)	0.128
cN1 (Y/N)	1.732(0.534–5.617)	0.360
Tumor length(≤3/>3cm)	1.013(0.285–3.602)	0.984
Bilateral disease (Y/N)	0.573(0.175–1.871)	0.356
CLNR (≤77.78%/>77.78%)	5.467(1.082–27.609)	0.040
Number of metastatic CLN (≤5/>5)	2.760(0.711–10.707)	0.142
Extent of thyroidectomy (TT/others)	1.440(0.409–5.069)	0.570
Lymph node dissection (Y/N)	1.004(0.558–1.805)	0.990
Hashimoto thyroiditis (Y/N)	1.147(0.284–4.634)	0.847
Multifocality (Y/N)	2.377(0.728–7.763)	0.152
Gross-ETE(Y/N)	1.346(0.401–4.516)	0.630
Micro-ETE(Y/N)	0.676(0.078–5.828)	0.722
Extranodal extension (Y/N)	3.200(0.829–12.354)	0.091
RAI(Y/N)	0.785(0.235–2.630)	0.695
pN1a	0.838(0.219–3.203)	0.796
pN1b	0.682(0.068–6.871)	0.745
LLNR (≤0.15%/>0.15%)	0.714(0.071–7.204)	0.775

CI, confidence interval; classic PTC, classic papillary thyroid cancer; cN1, clinically lymph node positive; CLNR, central lymph node ratio; TT, total thyroidectomy; CLN, central lymph node; gross-ETE, gross-extrathyroidal extension; micro-ETE, micro-extrathyroidal extension; RAI, radioactive iodine ablation.

Excluding cases without central lymph node dissection or detailed information on metastatic regional lymph nodes, our analysis included 85 patients with available CLNR data. Chi-squared and Mann-Whitney U tests were employed to compare clinicopathological variables between the high CLNR group (>77.78%) and the low CLNR group (≦77.78%), as detailed in **Table 3**. Significant disparities in gender, cN1, HT, and extrathyroidal extension were observed between the two groups (p<0.05).

**Table 3:** CLNR high group vs CLNR low group.

			percentage	p value
Association between CLNR and other characteristics				
CLNR vs gender	male	female		0.002
CLNR low	3	42	93.3%	
CLNR high	13	27	67.5%	
CLNR vs lymphovascular invasion	Invasion absent	Invasion present		1
CLNR low	40	5	11.1%	
CLNR high	35	5	12.5%	
CLNR vs cN1	cN1 absent	cN1 present		0.025
CLNR low	30	15	33.3%	
CLNR high	17	23	57.5%	
CLNR vs bilateral disease	bilateral absent	Bilateral present		0.192
CLNR low	34	11	24.4%	
CLNR high	25	15	37.5%	
CLNR vs distant metastasis	DM absent	DM present		1
CLNR low	43	2	4.4%	
CLNR high	38	2	5%	
CLNR vs multifocality	multifocality absent	multifocality present		0.077
CLNR low	33	12	26.7%	
CLNR high	22	18	45%	
CLNR vs HT	HT absent	HT present		0.001
CLNR low	29	16	35.6%	
CLNR high	38	2	5%	
CLNR vs extranodal extension	EE absent	EE present		0.103
CLNR low	39	6	13.3%	
CLNR high	29	11	27.5%	
CLNR vs ETE	ETE absent	ETE present		0.041
CLNR low	28	17	37.8%	
CLNR high	16	24	60%	
CLNR vs LLNM	LLNM absent	LLNM present		
CLNR low	5	15		1
CLNR high	1	26		
	CLNR high	CLNR low		
CLNR vs age	18(15–19)	17(16–19)		0.7091
	CLNR high	CLNR low		
CLNR vs tumor length	2.3(1.475–2.85)	2.3(1.5–3)		0.7913

cN1, clinically lymph node positive; CLNR, central lymph node ratio; DM, distant metastasis; HT, Hashimoto thyroiditis; EE, extranodal extension; ETE, extrathyroidal extension; RAI, radioactive iodine ablation; LLNM, lateral lymph node metastasis.

Multivariate regression analysis, detailed in **Table 4**, was conducted to delineate clinicopathological variables associated with the high CLNR group. This analysis revealed that cN1 status, HT, and gender were significantly linked to an elevated CLNR. Patients with cN1 faced a 3.161-fold increased risk compared to those without cN1. Those with HT were nearly 9.148 times more likely to fall into the high CLNR category than those without HT. Furthermore, male patients exhibited a significantly higher risk, with a 6.232 times greater likelihood than female patients.

**Table 4:** Multivariate Logistic Regression Analysis of Clinicopathological Variables to Predict High CLNR Group.

Factors	B	Wald	OR (95%CI)	p
**cN1**	1.151	4.681		0.030
Present			3.161(1.114–8.968)	
Absent			1	
**Hashimoto thyroiditis**	2.214	6.943		0.008
Present			1	
Absent			9.148(1.763–47.469)	
**Extrathyroidal extension**	-0.258	0.222		0.637
Present			1	
Absent			0.772(0.264–2.259)	
**Gender**	1.830	5.704		0.017
Male			6.232(1.388–27.976)	
Female			1	

## Discussion

Pediatric differentiated thyroid carcinoma (DTC) is recognized for its relatively high recurrence rate, which ranges from 13% to 20% (11, 13, 16, 17). Local recurrence often necessitates additional surgery, while distant recurrence can progress to a treatment-refractory state, necessitating long-term targeted therapy. Accurate prediction of recurrence postoperatively is thus crucial for determining follow-up schedules and selecting appropriate treatments. The lymph node ratio (LNR) has recently gained attention for its potential in predicting recurrence, with several studies suggesting it may better identify patients at risk for latent recurrence (18–20).

In our study, the rates of central and lateral lymph node metastasis were consistent with those reported by Thiesmeyer et al. (21). However, we found that neither lymph node status nor the number of metastatic lymph nodes were significant predictors of Recurrence-Free Survival (RFS) or recurrence in our cohort. This contrasts with our findings of the significance of central lymph node ratio (CLNR) when a cutoff value of 77.78% was applied. The CLNR significantly stratified RFS and was a notable predictor of recurrence in our univariate logistic analysis. Our findings are in line with two smaller studies (13, 22). and one involving 136 pediatric DTC cases, which found that an LNR above 0.34 significantly impacted persistence-free survival (23). Our research stands out as the first to use CLNR for predicting recurrence and stratifying RFS in pediatric DTC. The relevance of LNR is further underscored by its significance in adult DTC, as evidenced in a study of 325 PTC patients where an LNR above 0.3 was associated with a higher recurrence rate (24).

Upon comparing the high-CLNR group (CLNR > 77.78%) with the low-CLNR group (CLNR ≤ 77.78%), significant differences in gender, clinical lymph node positivity (cN1), Hashimoto Thyroiditis (HT), and extrathyroidal extension (ETE) were observed. Male patients, in particular, exhibited a higher CLNR, though gender was not a significant factor in our univariate analysis of recurrence. This is congruent with Guo et al.’s study of 217 pediatric DTC cases, which also found no significant correlation between gender and recurrence (16). However, two other studies have reported a direct correlation between male gender and recurrence and distant metastasis (10, 11), Considering that our cohort belongs to the same Asian population, we are inclined to believe that males may have a higher risk of recurrence.

The preoperative evaluation of cervical lymph node metastasis is critical, particularly given that the majority of patients in the high-CLNR group were clinically lymph node positive (cN1). While Sugino et al. have identified cN1 as an independent risk factor for recurrence (11), we did not find cN1 to be a significant predictor in our cohort, possibly due to our smaller sample size.

Patients with HT were found more frequently in the low-CLNR group, hinting at a potential protective effect against recurrence and lymph node metastasis. However, no significant correlation between HT and recurrence was observed in our univariate analysis, a finding supported by Yeker et al. (25). In contrast, Guo et al. identified HT as an independent risk factor for recurrence and lymph node metastasis (16), suggesting a complex relationship that warrants further investigation, especially in the pediatric DTC context.

Our study also revealed a positive correlation between the presence of ETE and CLNR, yet ETE did not show a significant relationship with recurrence in univariate analysis. This is consistent with some studies (16, 26) but contrasts with others, including Jeon et al.’s work, which identified ETE as an independent predictive factor for recurrence (10).

We advocate for preoperative evaluation of cervical lymph node metastasis using neck CT or MRI, followed by standard central compartment lymph node dissection with routine rapid frozen section pathological analysis. An elevated CLNR should prompt more extensive surgical intervention and a proactive postoperative regimen, including iodine-131 therapy, rigorous TSH level control, and comprehensive surveillance. These measures aim to minimize recurrence risk and facilitate the early detection of recurrent lesions, with timely surgical treatment advised for patients who experience recurrence.

This study, while contributing valuable insights into pediatric DTC, has several limitations. Its retrospective design and single-center data source may introduce selection bias. The limited sample size may affect the generalizability of the findings. Additionally, variations in long-term follow-up compliance could impact the assessment of recurrence. The study’s retrospective nature also precluded the collection of data on the size of metastatic lymph nodes, a factor noted to be significant in previous research and guidelines (22, 27). Despite these limitations, our research represents the largest cohort to date examining the clinical significance of CLNR in pediatric DTC.

## Conclusion

The present study has identified the Central Lymph Node Ratio (CLNR) as a robust prognostic indicator for recurrence in pediatric patients with Differentiated Thyroid Carcinoma (DTC). With a determined cutoff value of 77.78%, CLNR significantly stratified patients based on Recurrence-Free Survival (RFS), providing a more nuanced approach to risk assessment than the current American Joint Committee on Cancer (AJCC) TNM system. Our findings underscore the importance of incorporating CLNR into the clinical decision-making process for pediatric DTC management.

Patients characterized by a high CLNR exhibited a markedly increased risk of recurrence, with a hazard ratio of 5.467 times that of patients with a low CLNR. This group also presented with a notable prevalence of male gender, clinical lymph node positivity (cN1), Hashimoto Thyroiditis (HT), and extrathyroidal extension (ETE). These factors, in conjunction with a high CLNR, may inform a more aggressive treatment strategy, including the consideration of total thyroidectomy and the application of postoperative radioactive iodine ablation (RAI).

Our research advocates for a personalized approach to postoperative care, tailored to the individual patient’s risk profile. For those with a high CLNR, we recommend vigilant TSH suppression therapy and regular surveillance to promptly detect and address any recurrence. Furthermore, the prompt surgical management of recurrent lesions is emphasized to improve patient outcomes.

While our study contributes to the body of evidence supporting CLNR’s utility in pediatric DTC, it is not without limitations. The retrospective design, single-center data collection, and modest sample size may introduce biases and limit the generalizability of our findings. Additionally, the study did not account for the size of metastatic lymph nodes, a factor that may influence recurrence risk. Future research should aim to validate our findings in a larger, multicenter cohort and explore the interplay between CLNR and other established prognostic factors.

In conclusion, the introduction of CLNR as a prognostic tool represents a significant step forward in the personalized treatment of pediatric DTC. By refining our understanding of recurrence risk, CLNR has the potential to enhance clinical decision-making and ultimately improve patient care and outcomes in this vulnerable population.

## Data availability statement

The original contributions presented in the study are included in the article/supplementary material. Further inquiries can be directed to the corresponding author.

## Ethics statement

The studies involving humans were approved by First Affiliated Hospital of Guangxi Medical University Institutional Review Board. The studies were conducted in accordance with the local legislation and institutional requirements. Written informed consent for participation in this study was provided by the participants’ legal guardians/next of kin.

## Author contributions

CQ: Conceptualization, Data curation, Formal analysis, Funding acquisition, Investigation, Methodology, Project administration, Resources, Software, Supervision, Validation, Visualization, Writing – original draft, Writing – review & editing. SW: Resources, Writing – review & editing. JL: Funding acquisition, Project administration, Writing – review & editing.

## References

[1] Holmes L Jr. Hossain J Opara F. Pediatric thyroid carcinoma incidence and temporal trends in the USA (1973-2007): race or shifting diagnostic paradigm? ISRN Oncol. (2012) 2012:906197. doi: 10.5402/2012/906197 22530151 PMC3317016

[2] Kim J Gosnell JE Roman SA. Geographic influences in the global rise of thyroid cancer. Nat Rev Endocrinol. (2020) 16:17–29. doi: 10.1038/s41574-019-0263-x 31616074

[3] Qian ZJ Jin MC Meister KD Megwalu UC. Pediatric thyroid cancer incidence and mortality trends in the United States, 1973-2013. JAMA Otolaryngol Head Neck Surg. (2019) 145:617–23. doi: 10.1001/jamaoto.2019.0898 PMC6547136 31120475

[4] Vaccarella S Lortet-Tieulent J Colombet M Davies L Stiller CA Schüz J. Global patterns and trends in incidence and mortality of thyroid cancer in children and adolescents: A population-based study. Lancet Diabetes Endocrinol. (2021) 9:144–52. doi: 10.1016/s2213-8587(20)30401-0 33482107

[5] Bernier MO Withrow DR Berrington de Gonzalez A Lam CJK Linet MS Kitahara CM. Trends in pediatric thyroid cancer incidence in the United States, 1998-2013. Cancer. (2019) 125:2497–505. doi: 10.1002/cncr.32125 PMC6602875 31012956

[6] Francis GL Waguespack SG Bauer AJ Angelos P Benvenga S Cerutti JM. Management guidelines for children with thyroid nodules and differentiated thyroid cancer. Thyroid. (2015) 25:716–59. doi: 10.1089/thy.2014.0460 PMC4854274 25900731

[7] Dinauer C Francis GL. Thyroid cancer in children. Endocrinol Metab Clin North Am. (2007) 36:779–806. doi: 10.1016/j.ecl.2007.04.002 17673128

[8] Hay ID Gonzalez-Losada T Reinalda MS Honetschlager JA Richards ML Thompson GB. Long-term outcome in 215 children and adolescents with papillary thyroid cancer treated during 1940 through 2008. World J Surg. (2010) 34:1192–202. doi: 10.1007/s00268-009-0364-0 20087589

[9] Parvathareddy SK Siraj AK Qadri Z Ahmed SO DeVera F Al-Sobhi S. Lymph node ratio is superior to ajcc N stage for predicting recurrence in papillary thyroid carcinoma. Endocr Connect. (2022) 11. doi: 10.1530/ec-21-0518 PMC8859938 35044932

[10] Jeon MJ Kim YN Sung TY Hong SJ Cho YY Kim TY. Practical initial risk stratification based on lymph node metastases in pediatric and adolescent differentiated thyroid cancer. Thyroid. (2018) 28:193–200. doi: 10.1089/thy.2017.0214 29179646

[11] Sugino K Nagahama M Kitagawa W Ohkuwa K Matsuzu K Suzuki A. Cutoff age between pediatric and adult thyroid differentiated cancer: is 18 years old appropriate? Thyroid. (2022) 32:145–52. doi: 10.1089/thy.2021.0255 34549602

[12] Lebbink CA Links TP Czarniecka A Dias RP Elisei R Izatt L. European thyroid association guidelines for the management of pediatric thyroid nodules and differentiated thyroid carcinoma. Eur Thyroid J. (2022) 2022):11(6). doi: 10.1530/etj-22-0146 PMC9716393 36228315

[13] Rubinstein JC Dinauer C Herrick-Reynolds K Morotti R Callender GG Christison-Lagay ER. Lymph node ratio predicts recurrence in pediatric papillary thyroid cancer. J Pediatr Surg. (2019) 54:129–32. doi: 10.1016/j.jpedsurg.2018.10.010 30361076

[14] Bortz MD Kuchta K Winchester DJ Prinz RA Moo-Young TA. Extrathyroidal extension predicts negative clinical outcomes in papillary thyroid cancer. Surgery. (2021) 169:2–6. doi: 10.1016/j.surg.2020.04.003 32682508

[15] Tuttle RM Haugen B Perrier ND. Updated American joint committee on cancer/tumor-node-metastasis staging system for differentiated and anaplastic thyroid cancer (Eighth edition): what changed and why? Thyroid. (2017) 27:751–6. doi: 10.1089/thy.2017.0102 PMC5467103 28463585

[16] Guo K Qian K Shi Y Sun T Chen L Mei D. Clinical and molecular characterizations of papillary thyroid cancer in children and young adults: A multicenter retrospective study. Thyroid. (2021) 31:1693–706. doi: 10.1089/thy.2021.0003 34514877

[17] Rubinstein JC Herrick-Reynolds K Dinauer C Morotti R Solomon D Callender GG. Recurrence and complications in pediatric and adolescent papillary thyroid cancer in a high-volume practice. J Surg Res. (2020) 249:58–66. doi: 10.1016/j.jss.2019.12.002 31923715

[18] Kim HI Kim K Park SY Choe JH Kim JH Kim JS. Refining the eighth edition ajcc tnm classification and prognostic groups for papillary thyroid cancer with lateral nodal metastasis. Oral Oncol. (2018) 78:80–6. doi: 10.1016/j.oraloncology.2018.01.021 29496063

[19] Yip J Orlov S Orlov D Vaisman A Hernández KG Etarsky D. Predictive value of metastatic cervical lymph node ratio in papillary thyroid carcinoma recurrence. Head Neck. (2013) 35:592–8. doi: 10.1002/hed.23047 22730192

[20] Lee J Lee SG Kim K Yim SH Ryu H Lee CR. Clinical value of lymph node ratio integration with the 8(Th) edition of the uicc tnm classification and 2015 ata risk stratification systems for recurrence prediction in papillary thyroid cancer. Sci Rep. (2019) 9:13361. doi: 10.1038/s41598-019-50069-4 31527831 PMC6746784

[21] Thiesmeyer JW Egan CE Greenberg JA Beninato T Zarnegar R Fahey Iii TJ. Prepubertal children with papillary thyroid carcinoma present with more invasive disease than adolescents and young adults. Thyroid. (2022). doi: 10.1089/thy.2022.0098 36355601

[22] Back K Kim TH Lee J Kim JS Choe JH Oh YL. Optimal value of lymph node ratio and metastatic lymph node size to predict risk of recurrence in pediatric thyroid cancer with lateral neck metastasis. J Pediatr Surg. (2022). doi: 10.1016/j.jpedsurg.2022.07.010 35973863

[23] Xu Y Wang Y Zhang X Huang R Tian R Liu B. Prognostic value of lymph node ratio in children and adolescents with papillary thyroid cancer. Clin Endocrinol (Oxf). (2021) 95:649–56. doi: 10.1111/cen.14491 33914928

[24] Weitzman RE Justicz NS Kamani D Kyriazidis N Chen MH Randolph GW. How many nodes to take? Lymph node ratio below 1/3 reduces papillary thyroid cancer nodal recurrence. Laryngoscope. (2022) 132:1883–7. doi: 10.1002/lary.30084 35229306

[25] Yeker RM Shaffer AD Viswanathan P Witchel SF Mollen K Yip L. Chronic lymphocytic thyroiditis and aggressiveness of pediatric differentiated thyroid cancer. Laryngoscope. (2022) 132:1668–74. doi: 10.1002/lary.29908 PMC9033882 34687456

[26] Saliba M Alzumaili BA Katabi N Dogan S Tuttle RM Zoltan A. Clinicopathologic and prognostic features of pediatric follicular cell-derived thyroid carcinomas: A retrospective study of 222 patients. Am J Surg Pathol. (2022) 46:1659–69. doi: 10.1097/pas.0000000000001958 PMC9669120 36040037

[27] Haugen BR Alexander EK Bible KC Doherty GM Mandel SJ Nikiforov YE. 2015 American thyroid association management guidelines for adult patients with thyroid nodules and differentiated thyroid cancer: the American thyroid association guidelines task force on thyroid nodules and differentiated thyroid cancer. Thyroid. (2016) 26:1–133. doi: 10.1089/thy.2015.0020 26462967 PMC4739132

